# Hyaluronic Acid-Glycine-Cholesterol Conjugate-Based Nanoemulsion as a Potent Vaccine Adjuvant for T Cell-Mediated Immunity

**DOI:** 10.3390/pharmaceutics13101569

**Published:** 2021-09-27

**Authors:** Chih-An Lin, Hui-Min Ho, Parthiban Venkatesan, Chiung-Yi Huang, Yu-Jhen Cheng, Yu-Hsing Lin, Hua-Yang Lin, Tzu-Yang Chen, Ming-Hsi Huang, Ping-Shan Lai

**Affiliations:** 1 Ph.D. Program in Tissue Engineering and Regenerative Medicine, National Chung Hsing University, Taichung City 402204, Taiwan; fire0082@gmail.com; 2 National Institute of Infectious Diseases and Vaccinology, National Health Research Institutes, Miaoli City 350401, Taiwan; jasminho@nhri.edu.tw (H.-M.H.); anita9132@nhri.edu.tw (C.-Y.H.); yujhen@nhri.edu.tw (Y.-J.C.); 3 Department of Chemistry, National Chung Hsing University, Taichung City 402204, Taiwan; venkatesanwala@gmail.com (P.V.); cypanjimmy@gmail.com (T.-Y.C.); 4 Preclinical Development Research Department, Holy Stone Healthcare Co., Ltd., Hsinchu 114066, Taiwan; starlin@hshc.com.tw (Y.-H.L.); louislin@hshc.com.tw (H.-Y.L.)

**Keywords:** hyaluronic acid, cholesterol, squalene, nanoemulsion, vaccine adjuvants, PEG-free formulation, allergy

## Abstract

Clinical cases of allergic reaction that are due to excipients containing polyethylene glycol (PEG), a hydrophilic molecule commonly used in drug/vaccine formulations, has attracted much attention in recent years. In order to develop PEG-free adjuvants, we investigated the feasibility of natural ingredients in the human body such as hyaluronic acid in the form of hyaluronic acid-glycine cholesterol (HACH) conjugate as an excipient for vaccine formulation. Interestingly, HACH grafted with ~13 wt.% cholesterol has good water dispersity and can serve as an emulsifier to stabilize the squalene/water interfaces, yielding a milky white and isotropic emulsion (SQ@HACH) after being passed through a high-shear microfluidizer. Our results show that SQ@HACH particles possessed a unimodal average hydrodynamic diameter of approximately 190 nm measured by dynamic light scattering and exhibited good stability upon storage at 4 °C and 37 °C for over 20 weeks. The results of immunogenicity using a mouse model with ovalbumin (OVA) as the antigen revealed that SQ@HACH significantly enhanced antigen-specific immune responses, including the polarization of IgG antibodies, the cytokine secretions of T cells, and enhancement of cytotoxic T lymphocyte (CTL) activation. Moreover, SQ@HACH revealed lower local inflammation and rapidly absorbing properties compared with AlPO_4_ after intramuscular injection in vivo, indicating the potential functions of the HA-derived conjugate as an excipient in vaccine formulations for enhancement of T cell-mediated immunity.

## 1. Introduction

Vaccination is the most effective strategy to prevent or limit the severity of infection-associated syndromes by training the immune system to recognize and destroy the invading pathogens [1]. For safety issues, new-generation vaccine candidates usually employ a highly purified sub-portion of the pathogen as an antigen; however, these components often lack immunogenicity, thus necessitating adjuvants to facilitate the induction of adaptive immunity [2]. Currently, the most common salts for large-scale vaccination are aluminum-based mineral salts that mainly function as a depot to drive humoral immunity; however, they are usually limited by the weak stimulation of cell-mediated immunity [3,4]. Thus, it is important to make an inventory of suitable compounds for the development of an efficient adjuvant [5,6] with an acceptable profile of reactogenicity [7].

Several developmental adjuvants have been approved or authorized in prophylactic human vaccines for enhanced cell-mediated immunity, examples include squalene emulsions, saponins, Toll-like receptor (TLR) agonists, liposomes, and lipid nanoparticles [3]. While allergic reactions to vaccines are extremely rare clinically [8], excipients containing polyethylene glycol (PEG) are speculatively implicated in some severe cases such as anaphylaxis [9,10,11]. Currently, nanomaterial-based vaccines contain PEG-derivatives as a vaccine adjuvant (MF59 and AS03) and an excipient in COVID-19 vaccines, including licensed BNT162b2 and those under EUA (ChAdOx1 nCoV-19 and mRNA-1273) [12,13]. The presumption of PEG-induced allergic reaction was supported by the finding of complement activation-related pseudoallergy (CARPA) triggered by pre-existing anti-PEG IgG or IgE-mediated hypersensitivity reactions [14,15,16]. The Centers for Disease Control and Prevention has recommended the administration of the Pfizer/BioNTech and Moderna COVID-19 vaccines, and exclusion of any person who has a history of allergic reaction associated with any of the vaccine components, including PEG and PEG derivatives, such as excipient polysorbates as the stabilizer or emulsion adjuvant [14]. Therefore, it is necessary to develop a PEG-free adjuvant considering the potential risks of PEG allergy.

Hyaluronic acid (HA) is a water-soluble glycosaminoglycan consisting of repeating disaccharide units of *N*-acetyl glucosamine and glucuronic acid, it is naturally present in the human body [17]. HA has been widely used in biomedical applications, such as in joint cavity injections for the treatment of arthritis and in dermal fillers for aesthetic medicine because of its controlled biodegradability, biocompatibility in vivo, and ease of modification [17,18,19]. Although some clinical case studies have described HA as a dermal filler that may cause a foreign body reaction in the injection site with local inflammation and late granuloma formation [20,21], HA is still classified as a biomaterial of low immunogenicity and allergenicity [22,23]. The administration of high purity or non-animal source HA does not elicit cellular or humoral immunity, including anti-HA IgE and IgG production based on clinical or laboratory evidences [23,24]. HA has shown poor interaction with blood components, but not shown any sensory-motor or behavioral changes after epidural administration in rabbits; furthermore, HA does not produce genetic damage in Ames *Salmonella* assay [25]. These features reveal that HA possesses low cytotoxicity, neurotoxicity, and mutagenicity. Some studies showed that HA might inhibit the proliferation of several cells based on the molecular weight and dosage of HA [26,27,28,29]. Generally, high concentration of high molecular weight HA may inhibit the production of pro-inflammatory mediators [30] and remove inflammatory cells by inhibition of proliferation to reverse the effects of HA fragments [27]. Interestingly, low molecular weight HA or HA fragments have an opposite impact, inducing cell proliferation [26,31], promoting the production of inflammatory mediators [30], and successfully enhancing the activation of dendritic cells and T cells [32]. They can thus act as a potential biomaterial for the development of adjuvants based on their safety and immune-related regulatory functions [33,34]. Therefore, it is important to verify the relationship between molecular weight and immune activation of HA-based materials.

In this study, we designed a squalene-based nanoemulsion using a PEG-free emulsifier composed of natural HA, glycine, and cholesterol and then investigated the potential of HA-glycine-cholesterol (HACH) conjugates for the development of a vaccine adjuvant. The biocompatibility and possible immune regulation, including T cell and B cell responses to squalene-based HACH nanoemulsion mixed with ovalbumin (OVA) antigen protein after single-dose intramuscular injection in mice were evaluated in vivo and ex vivo.

## 2. Materials and Methods 

### 2.1. Materials

The 100 kDa hyaluronic acid (HA) was provided by Holy Stone Healthcare (Taipei, Taiwan). Cholesterol was purchased from Alfa Aesar (Tewksbury, MA, USA). *n*-(*tert*-Butoxycarbonyl) glycine (Boc-Gly-OH), ethyl cyanohydroxyiminoacetate (Oxyma), diisopropylcarbodiimide (DIC) and phosphotungstic acid (PTA) were purchased from Sigma-Aldrich (Burlington, MI, USA). Dicyclohexylcarbodiimide (DCC) and 4-dimethylaminopyridine (DMAP) were purchased from ACROS (Geel, Belgium). Dichloromethane (DCM) was purchased from Seedchem (Melbourne, Australia). Dimethyl sulfoxide (DMSO) was purchased from UniRegion Bio Tech (New Taipei, Taiwan). Trifluoroacetic acid (TFA) was purchased from Lancaster (Lancaster, Lancashire, UK).

### 2.2. Synthesis and Characterization of Hyaluronic Acid-Gly-Cholesterol (HACH) Conjugates

#### 2.2.1. Synthesis of Boc-Gly-Cholesterol

An amount of 1000 mg (2.59 mmol) of cholesterol and 480 mg (2.74 mmol) of Boc-Gly-OH were dissolved in 125 mL of DCM, and then 1070 mg (5.18 mmol) of DCC and 372 mg (3.04 mmol) of DMAP were added into the mixture solution and reacted for 12 h under a N_2_ atmosphere at room temperature. The reaction was traced by thin layer chromatography (TLC) to confirm that the coupling reaction had completed. After removing the solvent by rotary evaporation, the residues were purified by silica gel chromatography with a mobile phase mixture of hexane/acetone at 3/1. The structure of the product was identified by ^1^H-NMR spectroscopy (Agilent Technologies 400 MHz NMR, Santa Clara, CA, USA) and mass spectroscopy (TSQ Altis™ Triple Quadrupole Mass Spectrometer, Thermo Fisher Scientific, Waltham, MA, USA). ^1^H-NMR (400 MHz, CDCl_3_) δ 5.37 (d, *J* = 4.4 Hz, 1H), 4.99 (s, 1H), 4.68 (m, 1H), 3.87 (d, *J* = 5.3 Hz, 2H), 2.33 (d, *J* = 7.8 Hz, 2H), 2.01 (m, 2H), 1.95 (t, *J* = 4.5 Hz, 1H), 1.90−1.77 (m, 3H), 1.63−1.41 (m, 9H),1.45 (s, 9H), 1.40−1.23 (m, 5H), 1.20−1.03 (m, 8H), 1.01 (s, 3H), 0.91 (d, *J* = 6.6 Hz, 3H), 0.86 (dd, *J* = 6.6, 1.6 Hz, 6H), 0.67 (s, 3H); ESI-MS: C_34_H_58_NO_4_^+^ [M + H]^+^ 543.9 *m*/*z*.

#### 2.2.2. Deprotection of Boc-Gly-Cholesterol

An amount of 500 mg (0.92 mmol) of Boc-Gly-cholesterol was dissolved in 2 mL of DCM in an ice bath, and then 2 mL of TFA was added into the solution and stirred for 3 h under a N_2_ atmosphere at room temperature. The reaction was traced by TLC to check the completion of de-protection of Boc group. After de-protection, the mixture was neutralized with saturated NaHCO_3_ aqueous solution and the precipitate was filtered out and dried in vacuum to obtain the NH_2_-Gly-cholesterol white powder. The structure of NH_2_-Gly-cholesterol was identified by ^1^H-NMR spectroscopy (Agilent Technologies 400 MHz NMR) and mass spectroscopy (TSQ Altis™ Triple Quadrupole Mass Spectrometer). ^1^H-NMR (400 MHz, CDCl_3_) δ 5.37 (broad s, 1H), 4.68 (m,1H), 3.79 (broad s, 2H), 2.33 (m, 2H), 2.01 (m, 2H), 1.95 (t, *J* = 4.5Hz, 1H), 1.90–1.77 (m, 3H), 1.63–1.41 (m, 7H), 1.40–1.23 (m, 6H), 1.20–1.03 (m, 7H), 1.01 (s, 3H), 0.91 (d, *J* = 6.5 Hz, 3H), 0.86 (d, *J* = 6.5 Hz, 6H), 0.67 (s, 3H); ESI-MS: C_29_H_50_NO_2_^+^ [M + H]^+^ 444.2 *m*/*z*.

#### 2.2.3. Conjugation of HA and NH_2_-Gly-Cholesterol

An amount of 500 mg (1.25 mmol, 1.0 eq.) sample of HA (100 kDa) was first dissolved in a mixture of 70 mL of water and 90 mL of DMSO. A mixture containing 250 mg (1.11 mmol) of Oxyma and 113 mg (0.25 mmol, 0.2 eq.) of NH_2_-Gly-Cholesterol was dissolved in 10 mL of DMSO and then the resulting solution was added into the HA solution. The mixed solution was slowly added by 405 μL (2.58 mmol) of DIC and stirred for 24 h. The obtained solution was transferred into a 3500-MWCO dialysis bag and purified by sequential dialysis against DMSO/water (50/50, *v*/*v*), 0.3 M NaCl aqueous solution, and pure water. Finally, water was removed from the dialyzed product solution by freeze-drying to obtain HACH20. The different DS% of HACH was synthesized by adding corresponding equivalents of NH_2_-Gly-Cholesterol; HACH10 and HACH30 means 0.1 or 0.3 equivalent of NH_2_-Gly-Cholesterol for the HA conjugation, respectively. The conjugation ratio of HACH was determined by elemental analysis (Elementar vario EL cube, Langenselbold, Hesse, Germany).

The DS% of HACH was analyzed by an elemental analyzer (EA) and calculated using the following Formula (1):

(1) $$\;\mathrm{DS}\%=\frac{R_{y}-R_{0}}{R_{100}-R_{0}}\times 100\%$$

where *R_y_* is the C/N ratio of HACH, and *R*_0_ and *R*_100_ represent the C/N ratios of unmodified HA and theoretical completely modified HACH (100%), respectively.

### 2.3. Preparation and Characterization of HACH-Stabilized Squalene Emulsion (SQ@HACH)

An amount of 100 mg of HACH20 was dissolved in 9.5 mL of sodium citrate solution (pH = 6.5, 10 mM), and then 500 μL of squalene was added into the HACH solution. The resulting solution was pre-mixed in a test tube rotator at 500 rpm for 1 h and then homogenized through a high-pressure microfluidizer operated at 20,000 psi (Nanolyzer N2, Gogene Corporation, Hsinchu County, Taiwan). To evaluate the particle size and stability of SQ@HACH, aliquots of the SQ@HACH emulsion were loaded in 1.5-mL Eppendorf tubes and stored separately at 4 °C and 37 °C. At predetermined time points, the appearance and particle sizes of the emulsion were recorded. The particle size and polydispersity index (PDI) of HACH20 and SQ@HACH in water solutions were measured by dynamic light scattering (DLS, Malvern Zetasizer Nano ZS90, Malvern, UK). The HACH20 and SQ@HACH were stained by PTA negative staining and their morphology was observed by transmission electron microscopy (TEM, JEOL JEM-1400 electron microscopy, Tokyo, Japan).

### 2.4. Animals and Ethics Statement

Female C57BL/6 mice, 6–12 weeks old, were obtained from the National Laboratory Animal Center (Taipei, Taiwan). All animals were housed at the Animal Center of National Health Research Institutes (NHRI) and maintained in accordance with the institutional animal care protocol. All of the animal studies were approved by the animal committee of the NHRI (NHRI-IACUC-107149).

### 2.5. Immunization In Vivo and Tissue Sample Preparation

#### 2.5.1. Immunization Schedule

C57BL/6 mice (three mice per group) were immunized by intramuscular injection at the quadriceps (50 μL each leg) with a total of 50 μg of OVA in sodium citrate solution (pH = 6.5, 10 mM) in the absence or presence of SQ@HACH (squalene content: 5 μL). An amount of 50 μg OVA with the aluminum mineral salt adjuvant (AlPO_4_, aluminum content: 30 μg, BRENNTAG, Vejle, Denmark) was used as control adjuvant. These samples were gently homogenized by rotation prior to injection.

#### 2.5.2. Preparation of Muscular Tissues at the Injection Sites and Splenocyte Samples

Fourteen days post-immunization, both the quadriceps and spleen were harvested. The quadriceps tissues were stored in PBS buffer, washed with saline, fixed in formalin, embedded in paraffin and cut into thick sections on slides (4 μm). The slides were stained with hematoxylin and eosin (H&E).

The spleens were temporarily pooled in a sterile lymphocyte culture medium to avoid cell death. The lymphocyte culture medium (LCM) contained 900 mL of RPMI 1640, 100 mL of FBS, 25 mL of HEPES (25 mM), and 50 µL of *β*-mercaptoethanol (150 µM). To obtain splenocyte samples, the spleen was gently pressed with the plunger seal of a 5-mL syringe on a 70-µm cell strainer into a 50-mL tube and washed through a cell strainer with RPMI 1640 containing 10% (*v*/*v*) FBS. Then, the washing buffer was removed by centrifugation at 1200 rpm for 5 min at 4 °C. The cell pellet was resuspended in 5 mL red blood cell lysis buffer (dilution from RBC lysis buffer (10×), Biolegend, Lot: B166991) and incubated in an ice bath. Then, 30 mL of cold PBS was added for lysis quenching, and the supernatant was removed by centrifugation at 1200 rpm for 5 min at 4 °C. Finally, the cell pellets were gently resuspended in LCM. The cell solution was counted and diluted to the desired cell concentrations.

#### 2.5.3. Preparation of the Sera Samples

C57BL/6 mice (six mice per group) were immunized on the same schedule as described in Section 2.5.1. Serum samples from immunized mice were collected by withdrawing submandibular blood at predetermined times, and they underwent a centrifugation at 7500 rpm for 15 min.

### 2.6. MRNA Expression of T Cells

A total of 5 × 10^6^ cells/mL splenocytes from immunized mice were seeded in 24-well plates and re-stimulated with or without OVA (low-endo; the final concentration of OVA was 10 μg/mL). After 24 h, the total RNA of splenocytes was extracted using TRIzol reagent (Invitrogen, Waltham, MA, USA) according to the manufacturer’s instructions. An amount of 1 μg of total RNA was used for cDNA synthesis with oligo (dT). Real-time PCR was carried out using the Light Cycler 480 II (Roche, Basel, Switzerland). The geometric mean of the housekeeping gene *β*-actin was used as an internal to normalize the variability in expression levels, and it was analyzed using the 2^−ΔΔCT^ method.

### 2.7. Cytotoxic T Lymphocyte-Mediated Killing Activity

A total of 5 × 10^6^ cells/mL splenocytes from immunized mice were seeded in 24-well plates and re-stimulated with or without 1 μg/mL H-2K^b^ OVA peptide (OVA_257–264_, SIINFEKL, MBL, Code No.: TS-5001-P) for 24 h and detected by cell staining using anti-CD8-PE (MBL, Code No.: D271-5), anti-CD19-PE-Cy7 (Biolegend, Cat No.: 115520) and Live/Dead-FITC. The stained OVA-specific CD8+ T cells (gating strategy: total cells/live cells/CD19-/CD8+) were analyzed by flow cytometry. The FACS data were analyzed and calculated by Flow Jo software (Version v10, Ashland, OR, USA), and the results are shown as the mean fluorescence intensity and population percentage.

### 2.8. IgG and IgG Subtype Titers

The titers of OVA-specific IgG and IgG subtypes were measured using goat anti-mouse IgG H&L (HRP) (Abcam, Cambridge, UK), goat anti-mouse IgG1 (HRP) (Abcam, Cambridge, UK) and goat anti-mouse IgG2a (HRP) (Abcam, Cambridge, UK). OVA-specific IgG tracking was performed with the serial dilution method for analysis by ELISA; the titers were read as the UV absorbance signals, which twice exceeded the background value.

### 2.9. Statistical Analysis

The IgG antibodies are presented as geometric mean titers with 95% confidence intervals; the significance among the groups was determined by Tukey’s one-way ANOVA tests to perform comparisons, followed by post hoc tests, and a *p*-value < 0.05 was considered to indicate a statistically significant difference. The physicochemical characteristics and T cell responses are expressed as the mean ± standard deviation (SD).

## 3. Results and Discussion

### 3.1. Synthesis and Characterization of HACH

**Figure 1:**
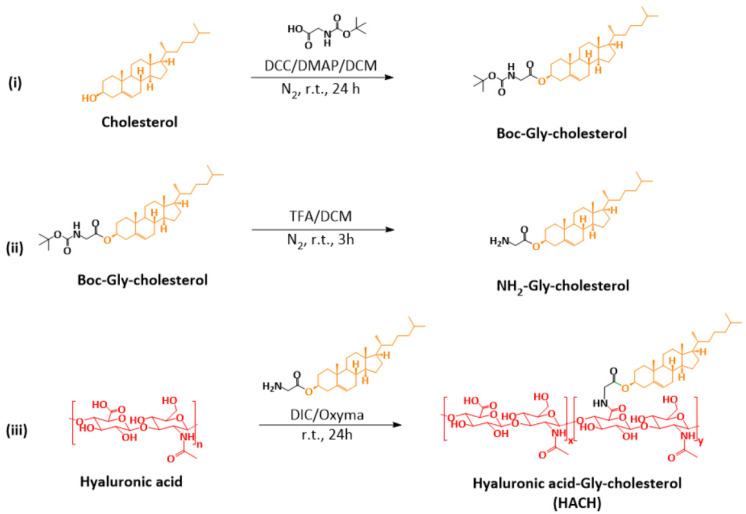
Synthetic pathway of HACH. In the first step, cholesterol was modified with Boc-Gly-OH by DCC/DMAP esterification. In the second step, the Boc group was removed under acidic conditions by TFA to produce NH_2_-Gly cholesterol. In the final step, NH_2_-Gly-cholesterol was conjugated to the carboxylic group of HA by DIC/Oxyma activation. (**i**) Modification of cholesterol; (**ii**) deprotection of Boc-Gly cholesterol; (**iii**) conjugation of Gly cholesterol on HA.

Cholesterol, a natural molecule that stabilizes cell membranes as well as liposome carriers, was used to confer an amphiphilic property to HA [35]; the cholesterol molecule represents the hydrophobic part of HACH conjugates and the synthetic procedures are shown in Figure 1. In the first step (i), the cholesterol was modified by Glycine-Boc using Steglish esterification to generate Boc-Gly cholesterol, and then (ii) the Boc group was removed by TFA/DCM. As shown in Figure S1, the characteristic peaks of cholesterol (methyl groups at 0.67, 0.86, 0.91, and 1.01 ppm) and *tert*-butyl group (Peak h, 1.45 ppm, singlet, 9H) were observed in Boc-Gly cholesterol (Figure S1a) and the Boc peak at 1.45 ppm disappeared after TFA incubation (Figure S1b), indicating a successful de-protection process for obtaining NH_2_-Gly-cholesterol. The molecular weight of NH_2_-Gly-cholesterol was also confirmed by mass spectroscopy. 

HACH with a glycine linker was synthesized using DIC/oxyma as an amide coupling agent to graft NH_2_-Gly-cholesterol onto HA in a DMSO/H_2_O co-solvent system (iii). After dialysis and lyophilization, the cotton-like HACH product was obtained. It is noticed that the characteristic peaks of cholesterol (four peaks of methyl groups at 0.67, 0.86, 0.91, and 1.01 ppm) in HACH were not observed in the ^1^H-NMR spectrum (Figure S1c), this finding suggested the presence of microphase-separated structure of HACH. Fieber et al. used self-diffusion NMR spectroscopy and relaxometry to explain this phenomenon of the hydrophobic core. They found that the rigidity of the hydrophobic core of the macromolecular structure would decrease the T1 and T2 relaxation times, which enabled the NMR signal to be undetectable [36]. In HACH, it is speculated that the original hydrophilic HA chain exhibits high flexibility and mobility, and this property is influenced by cholesterol conjugation, resulting in an increase of mobility from the terminal side of cholesterol groups toward the HA backbone. Therefore, the signal peaks of cholesterol cannot be easily detected by ^1^H-NMR in HACH.

The theoretical carbon to nitrogen (C/N) ratio of HA alone or HACH with 100% degree of substitution (DS) was 14 and 21.5, respectively. To determine the percentage of DS of cholesterol of HACH, we employed EA to investigate the C/N ratio of free HA and HACH and then calculated the DS ratio of HACH using Formula (1). As shown in Table 1, the actual C/N of HA measured by EA was 14.0, this value was consistent with the theoretical C/N ratio of HA and indicated the reliability of EA analysis in the determination of C/N ratio of HA or HA-drug conjugates. The C/N of HACH10 was 14.7 analyzed by EA and the DS ratio was calculated as 9.1% that was closed to the estimated DS ratio of 10 %. HACH20 revealed a C/N ratio of 15.0, and DS ratio of 13.3% even though the DS ratio of HACH was estimated at 20 % for the conjugation process. If the estimated DS ratio was increased to 30%, the measured C/N ratio was increased to 15.6, and the DS ratio was 21.2%; however, HACH30 would become insoluble in an aqueous solution and thus could not be utilized for further applications. Thus, HACH20 was utilized for further emulsion and biomedical investigations.

### 3.2. Self-Assembling Properties of HACH without or with Squalene

**Figure 2:**
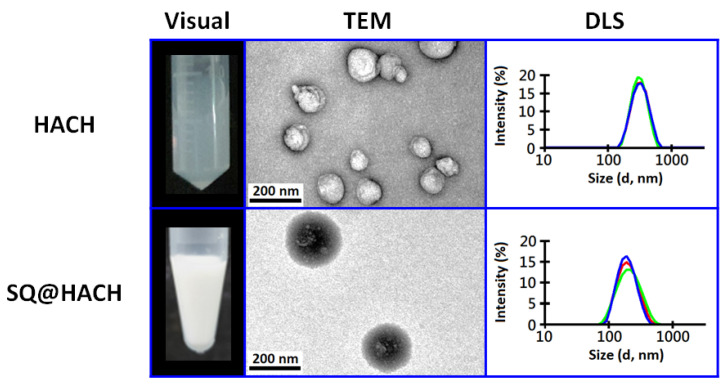
Visual appearance, TEM images and particle size distribution of HACH and SQ@HACH. The Size distribution was measured by DLS under 100-fold dilution of sample stock in water; colorful lines represent three different measurement results.

As shown in Figure 2, the resulting HACH20 product (DS 13.3%) is cloudy and well dispersed in an aqueous solution. In the TEM image, we found that HACH could self-assemble to form particles that were approximately 100 nm, whereas the particle size of HACH20 analyzed by DLS was 301.5 ± 3.2 nm. This difference in size was suggested to be caused by hydrophilic swelling of hyaluronic acid on the surface of the HACH20 particles in aqueous conditions. It was also found that the HACH30 product with a higher DS ratio gave rise to an unsatisfactory water-dispersibility that might be due to the high hydrophobicity from the increased cholesterol content. 

In order to evaluate the potential of amphiphilic HACH as an emulsifier for stabilizing the oil/water interfaces, HACH20 were mixed with 5% squalene and then passed through a high-pressure microfluidizer. As shown in Figure 2, an isotropic emulsified formulation (named SQ@HACH) was obtained after homogenization of the squalene/HACH20/citrate buffer. The TEM images reveal that SQ@HACH spherical particles were composed of some squalene droplets (bright core) surrounded by the amphiphilic HACH (dark shell). The results of DLS of SQ@HACH show a uniform size of around 190 ± 2 nm and a polydispersity of 0.136 ± 0.027. Compared with the self-assembled HACH in aqueous solution, the smaller-sized SQ@HACH particles might be a result of the hydrophobic interactions between squalene and hydrophobic segment of HACH after passing through a microfluidizer. It is noticed that the emulsification process predominated the particle size of SQ@HACH (Figure S2) whereas free HA solutions with squalene immediately showed phase separation under the same process (Figure S3), indicating that HACH20 successfully stabilized the squalene/water interface. We also evaluated the emulsifying ability of low DS ratio of HACH10 and found that this was insufficient to form a stable squalene nanoemulsion in spite of its excellent water-dispersibility. Thus, squalene nanoemulsion using 13.3% DS ratio of HACH20 was employed for the following studies.

### 3.3. Long-Term Storage of SQ@HACH

**Figure 3:**
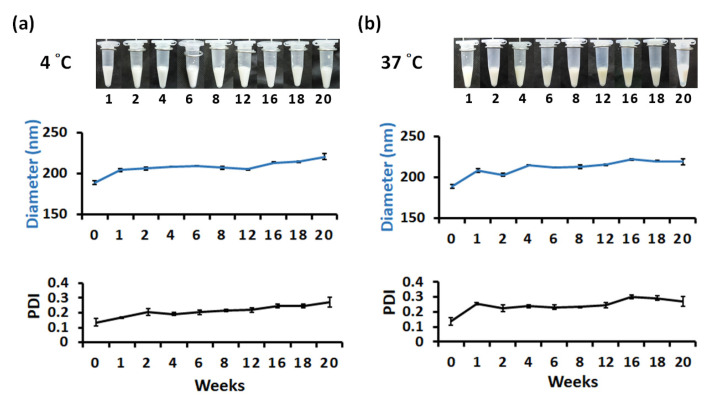
The changes of visual appearances, particle size and polydispersity index (PDI) of SQ@HACH upon storage at (**a**) 4 °C for 20 weeks and (**b**) 37 °C for 20 weeks. The diameter and PDI of SQ@HACH (100-fold dilution) were measured by DLS. (*n* = 3, mean ± SD).

SQ@HACH was stored in a refrigerator at 4 °C and an incubator at 37 °C to mimic the stability of SQ@HACH in cold chain storage and human body conditions, respectively. The physical appearance, particle size, and polydispersity index of SQ@HACH were monitored for at least 20 weeks. As shown in Figure 3a, the physical appearance of SQ@HACH did not change significantly over 20 weeks when stored at 4 °C. 

A significant increase in particle size of SQ@HACH from 190 nm to 200 nm was observed during the first 2 weeks, which might be due to coalescence induced by thermodynamic instability [37,38]. Then, SQ@HACH appeared to be stable in size from week 2 to week 16. After 16 weeks, the particle size slightly increased due to instability, during which the PDI increased in a similar trend.

Upon storage at 37 °C, the physical appearance of SQ@HACH did not obviously change during the first 12 weeks, but it gradually changed from a milky white emulsion to a light-yellow emulsion after 12 weeks (Figure 3b). This gradual color change may be assumed to be caused by the accelerated oxidation of squalene at high temperatures [39]. A significant increase in particle size was detected from approximately 190 nm to 215 nm during the first 4 weeks. Next, the particle size was maintained in the range of 210−220 nm between week 4 and week 16. After 16 weeks, the particle size remained at approximately 220 nm; however, the PDI slightly increased during this period. At week 20, slight emulsion delamination occurred, and both the particle size and PDI increased.

These data reveal that SQ@HACH has good stability of 4 °C storage for at least 16 weeks, indicating the advantage of SQ@HACH for last-mile transportation. This feature may be considered as a favorable storage strategy that may not only reduce the cost of cold chain transportation but also expand the availability of medicines (drugs or vaccines) to areas devoid of cold chain facilities.

### 3.4. Histological Examination of the Injection Site

**Figure 4:**
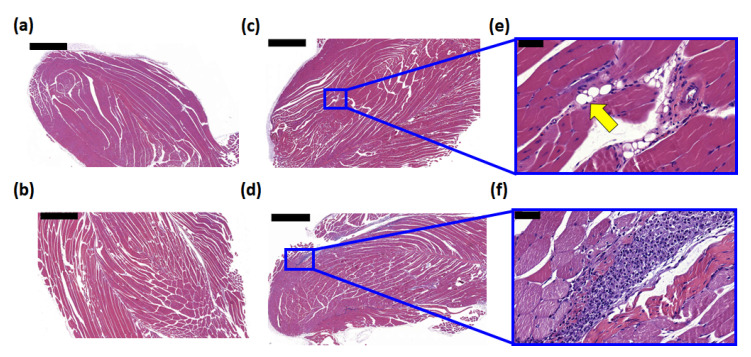
Histological analysis of the injection site in the quadriceps muscles. C57BL/6 mice were immunized with 100 μL of (**a**) 10 mM sodium citrate solution, pH = 6.5, (**b**) OVA 50 μg, (**c**,**e**) OVA 50 μg with SQ@HACH, and (**d**,**f**) OVA 50 μg with AlPO_4_ (aluminum content 30 μg) at quadriceps. The quadriceps muscle tissues were removed on day 14 and stained by H&E staining. The muscle sections were observed by microscopy at magnification (10× and 40×). Scale bar: 1000 μm (**a**–**d**) and 50 μm (**e**,**f**). The yellow arrow indicates the vesicles of SQ@HACH.

To assess the cell infiltration and absorbance of HACH and SQ@HACH at the injection site, C57BL/6 mice were divided into 4 groups including a control (0.1 mM sodium citrate buffer, pH = 6.5), OVA alone, OVA with SQ@HACH, and OVA with a conventional AlPO_4_ adjuvant; the latter was treated for comparison. Fourteen days after injection, the immunized mice were euthanized, and the muscle tissues of the injection site were removed for histological analysis. The H&E-stained quadriceps muscle section is shown in Figure 4. There was no cell infiltration in the buffer control and OVA-alone groups (Figure 4a,b). The AlPO_4_ adjuvant was soluble in water, and the residue could not be observed in the examination; however, the infiltration of immune cells from the muscle tissue showed that OVA with the AlPO_4_ adjuvant induced a greater degree of local inflammation than SQ@HACH (Figure 4e). Similar to the OVA alone, we did not observe cell infiltration in the OVA with SQ@HACH group (Figure 4f). The SQ@HACH particles were almost absorbed, and the residue materials were clustered into several 10–20 μm vesicles, with a few recruited cells remaining. Overall, SQ@HACH shows tolerance following intramuscular injection in mice, a feature that can be further used in biomedical applications.

### 3.5. Serum IgG Antibodies

**Figure 5:**
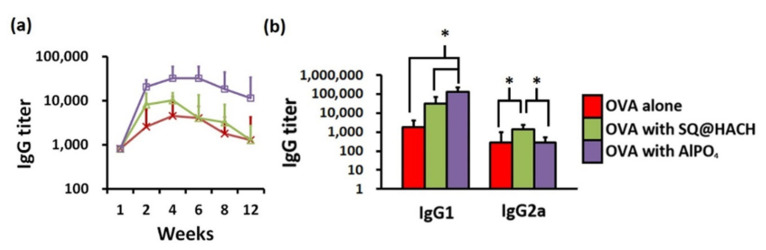
The impact of adjuvants on driving OVA-specific IgG antibodies in mice (*n* = 6 per group) treated with OVA alone, OVA with SQ@HACH and OVA with AlPO_4_ for 12 weeks. (**a**) IgG (**b**) IgG subtypes. The serum samples from immunized mice were collected for analysis by ELISA. The IgG subtypes were further analyzed in Week 4 by ELISA. The data are shown as the GMT ± 95% C.I. * *p* < 0.05.

The production of OVA-specific IgG antibodies is one of the main evaluation criteria of vaccine efficacy for humoral immunity. To investigate the impact of SQ@HACH on B cell-mediated IgG secretion, blood was regularly collected from the immunized mice, and the serum was traced by an ELISA assay. As shown in Figure 5a, vaccination with OVA and SQ@HACH induced a geometric mean titer (GMT) of 8000 at week 2, and the highest GMT of 10,000 was titrated at week 4; beyond, this titer rapidly diminished after week 6 and showed the same GMT level as the OVA alone, but it should be noted that the humoral immunity was induced by a single injection. These results correlate with the results of histological analysis. SQ@HACH was quickly absorbed within a few weeks and did not cause long-term inflammation of the injection site. On the other hand, the AlPO_4_ group induced a GMT of 32,000 at week 4, and it maintained a GMT for more than 10,000 for at least 12 weeks. 

### 3.6. Modulation of the Adjuvant-Mediated T Cell Activation

**Figure 6:**
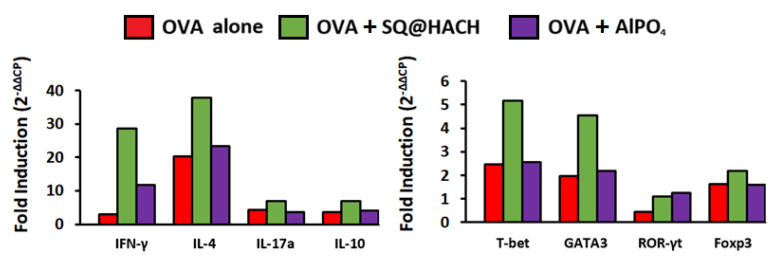
T cell-related mRNA quantification. Pooled splenocytes from immunized mice were obtained at day 14 after vaccination and re-stimulated with or without ovalbumin for 24 h. The quantification of T cell-related mRNA expression by RT-PCR for IFN-γ, IL-4, IL-17a, IL-10, T-bet, GATA3, ROR-γt, and Foxp3. The fold induction is the ratio of the expression values with ovalbumin to those without ovalbumin. Results are expressed as the geometric mean of the housekeeping gene beta-actin which was used as an internal control to normalize the variability in expression levels.

To investigate the effects of SQ@HACH on the subsequent signal transduction of T cells, splenocytes from immunized mice were collected for primary culture without or with OVA stimulation. After 24 h of incubation, mRNA expression was detected by qPCR, and the housekeeping gene beta-actin was used as an internal control for normalization. The fold induction was calculated from the ratio of the expression levels with OVA re-stimulation to those without stimulation. The data show that immunization by OVA adjuvanted with SQ@HACH increased the mRNA expression of IFN-γ (a Th1-type cytokine) and IL-4 (a Th2-type cytokine) compared with the OVA immunization without the formulation (Figure 6). These results are consistent with the mRNA expression of the specific key transcription factors T-bet (Th1) and GATA3 (Th2). We also detected the increased generation of IgG2a antibodies in the mice that received SQ@HACH-formulated OVA after 4 weeks of vaccination (Figure 5b), which predominated Th1 polarization. These findings suggest that SQ@HACH can enhance the related pathways of Th1 and Th2 cells simultaneously during the re-stimulation of OVA. It is important to note that the splenic cytokine secretion responses and serum IgG2a antibodies induced by OVA with AlPO_4_ adjuvant were the same as those induced by OVA alone, which is in agreement with the literature [40].

HA is known to play multifunctional roles in innate and adaptive immune responses via different receptors on immune cells [41]. HA-decoration can enhance cellular uptake efficiency by HA-CD44 receptor-mediated endocytosis in DCs [42,43], and it can also enhance the antigen delivery to lymph nodes which is likely to rely on the LYVE-1 receptors present on the endothelium of lymphatic vessels [44,45]. The above mechanisms facilitate the potential applications of HA or HA-derivatives as vaccine adjuvant candidates. 

In addition, low molecular weight (LMW) HA fragments act as damage-associated molecular pattern molecules (DAMPs) [46,47], which are recognized by TLRs of a wide range of immune cells, especially TLR-4 triggered DC maturation [48,49], and TLR-2 engagement on DCs promotes effector and memory T helper (Th) cell response [47,50].

Thus, we speculate the generation of LMW HA fragments from the degradation of HACH can further activate DCs and enhance antigen recognition and antigen presentation to Th cells. Of note, Pietà et al. also report similar results on HA-bioconjugated OVA, and they indicate that HA also stimulates a T cell immunity, especially Th1 immune response, and leads to the generation of an OVA-specific cytotoxic response [51]. Detailed investigations are ongoing at our laboratory.

### 3.7. Evaluation of the OVA-Specific Splenic Cytotoxic T Lymphocyte Response

**Figure 7:**
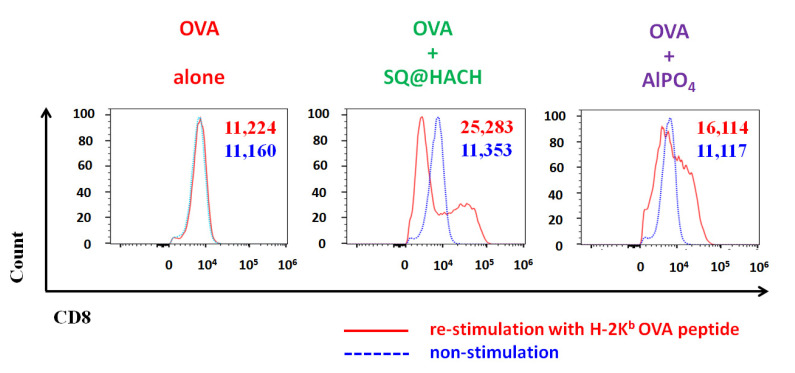
Activation of antigen-specific cytotoxic T lymphocytes. The splenocytes from immunized mice were obtained on day 14 after vaccination. Flow cytometric histograms and mean fluorescence intensity of CD8+ T cells were shown as re-stimulation for 24 h with H-2K^b^ OVA peptide (OVA_257–264_, SIINFEKL) (red) or with medium only (blue), respectively.

Cytotoxic T lymphocyte (CTL) immunity functions as the main defense against cancer and viral infections. In order to study whether SQ@HACH generated antigen-specific CTLs, splenocytes from immunized mice were collected and re-stimulated for 24 h with H-2K^b^ OVA peptide (OVA_257–264_, SIINFEKL), a peptide sequence associated with the phagocytic activity of DCs that can bind to the H-2K^b^ of MHC class I [52]. As shown in Figure 7, there was no difference in CD8+ cells with OVA_257–264_ re-stimulation (MFI of 11,224) and those without stimulation (MFI of 11,160) in the OVA-alone group. Interestingly, for the mice that received SQ@HACH-adjuvanted OVA, the MFI increased to 25,000 under the stimulation of OVA_257–264_ peptide, which was higher than the detected level (MFI of 16,000) in the AlPO_4_ group. The higher MFI value from the SQ@HACH-adjuvanted group’s splenocytes has a higher CD8 expression cell population than other groups when re-stimulated with OVA_257–264_ peptide. The upregulated CD8 expression can increase CTL activity against viral infections, a feature also found in COVID-19 patients recently [53]. In summary, the SQ@HACH-adjuvanted vaccine can enhance antigen-specific CTL immunity. An antigen-specific CTL response will be activated to attack when recognizable viral infections and cancer cells are encountered [54,55].

It has been reported that HA from the extracellular matrix is transiently broken down to LMW HA upon local inflammation during the early stage of infection [46], and LMW HA can directly activate TLRs [47]. Among these TLRs, TLR-2 plays the key role in enhancing effector and memory CD8 T cells response [56]. Thus, despite limited understanding of the role of SQ@HACH in CD8 T cell response, we speculated that the degradation of HACH could play a similar role as LMW HA to activate the proliferation of the effector CD8 T cells and further increase the probability of producing memory CD8 T cells. In the future, we will further explore the mechanisms in greater detail on how the SQ@HACH enhances CTL response.

Currently, nanotechnology is already applied to the design of vaccines. The importance of nanovaccines has been raised as a potent vaccine design to overcome the disadvantages of traditional vaccines [34,57]. These nanovaccine designs allow the encapsulation of antigen and adjuvant together to enhance immune stimulation, protect the antigens [58], and promote DCs maturation [59]. Furthermore, coating HA on the nanomaterial surface can also increase the permeability of the lymphatic vessels through lymphatic vessel endothelial receptor 1 (LYVE-1) [60], leading to the delivery of antigen to the lymph nodes. In this study, HACH was used a surfactant for the preparation of squalene nanoemulsion. The so-obtained SQ@HACH nanoemulsion can directly mix with antigen to evaluate the efficacy of SQ@HACH on immune stimulation. However, the function of antigen encapsulation has not yet been considered. Further investigations are warranted to tune the encapsulating parameters and manufacturing process on the design of HACH-based nanovaccines.

The above results indicate that the absorbance of SQ@HACH may reduce inflammation at the injection site. Nevertheless, SQ@HACH can accurately induce the immune activation of adjuvant-mediated OVA-specific splenic T cells in a single shot, especially the Th1-related CTL response. Moreover, SQ@HACH has great potential in vaccine storage and delivery due to its favorable stability. We will further investigate the relevant mechanisms between the SQ@HACH structural control and immune regulation in order to optimize the vaccine formulation and test its efficacy in the treatment of immune dysfunctions and infectious diseases.

## 4. Conclusions

In this study, we designed and synthesized a hyaluronic acid-glycine-cholesterol nanocomposite, HACH, as an alternative emulsifier to the commonly used PEG-derivatives to avoid allergy risk. The amphiphilic structure enables HACH emulsification to stabilize the squalene/water interfaces, thus rendering uniform nanoemulsions (SQ@HACH) with an average diameter of approximately 190–220 nm. SQ@HACH showed good stability during storage and tolerance in vivo. As a vaccine adjuvant, SQ@HACH can enhance T cell cytokine-related mRNA expression and bias the trends of IgG subtype titers. Under stimulation with an OVA epitope peptide, splenocytes from mice that received SQ@HACH-adjuvanted OVA had high CD8+ expression. Taken together, SQ@HACH has good potential as a candidate adjuvant for the development of vaccines against infectious diseases and cancer; moreover, the stability under mild storage condition is conducive to the distribution of vaccines.

## Figures and Tables

**Table 1:** C/N ratio and DS% of the cholesterol on HA.

	C Element (Mole%) ^a^	N Element (Mole%) ^a^	C/N ^b^	DS(%) ^c^
HA	3.125	0.223	14.0	-
HACH10	3.314	0.226	14.7	9.2
HACH20	3.143	0.210	15.0	13.3
HACH30	3.361	0.216	15.6	21.2

^a^: Determined by elemental analysis. ^b^: Molar ratio of carbon to nitrogen. ^c^: DS = (*R_y_* − *R*_0_)/(*R*_100_ − *R*_0_) × 100%.

## Data Availability

The data presented in this study are available on request from the corresponding author.

## Supplementary Materials

The following are available online at https://www.mdpi.com/article/10.3390/pharmaceutics13101569/s1, Figure S1: Structural identification: ^1^H NMR (CDCl_3_, 400 MHz) and mass spectrums (ESI-MS) of (a) Boc-Gly-cholesterol, (b) NH_2_-Gly-cholesterol; ^1^H NMR spectrum (D_2_O/d6-DMSO, 400 MHz) of (c) HACH20, Figure S2: Size distribution of SQ@HACH with different passages: (a) 1 passage, (b) 2 passages, and (c) 3 passages through a microfluidizer, Figure S3: Appearance of (a) SQ@HA and (b) SQ@HACH. 5% squalene was added to the solution containing 1% HA and 1% HACH, homogenized and stood for 1 h.
