# Supplementary material for: Hyaluronic Acid-Glycine-Cholesterol Conjugate-Based Nanoemulsion as a Potent Vaccine Adjuvant for T Cell-Mediated Immunity
Source: Pharmaceutics. 2021 Sep 27;13(10):1569. doi: 10.3390/pharmaceutics13101569 (PMC8539354; doi:10.3390/pharmaceutics13101569)
Supplement: Supplementary file 1 [file pharmaceutics-13-01569-s001.zip › pharmaceutics-1350208-supplementary.pdf]

pharmaceutics

MDPI

Supplementary Materials: Hyaluronic Acid-Glycine-Cholesterol Conjugate-Based Nanoemulsion as a Potent Vaccine Adjuvant for T Cell-Mediated Immunity

Chih-An Lin, Hui-Min Ho, Parthiban Venkatesan, Chiung-Yi Huang, Yu-Jhen Cheng, Yu-Hsing Lin, Hua-Yang Lin, Tzu-Yang Chen, Ming-Hsi Huang * and Ping-Shan Lai *

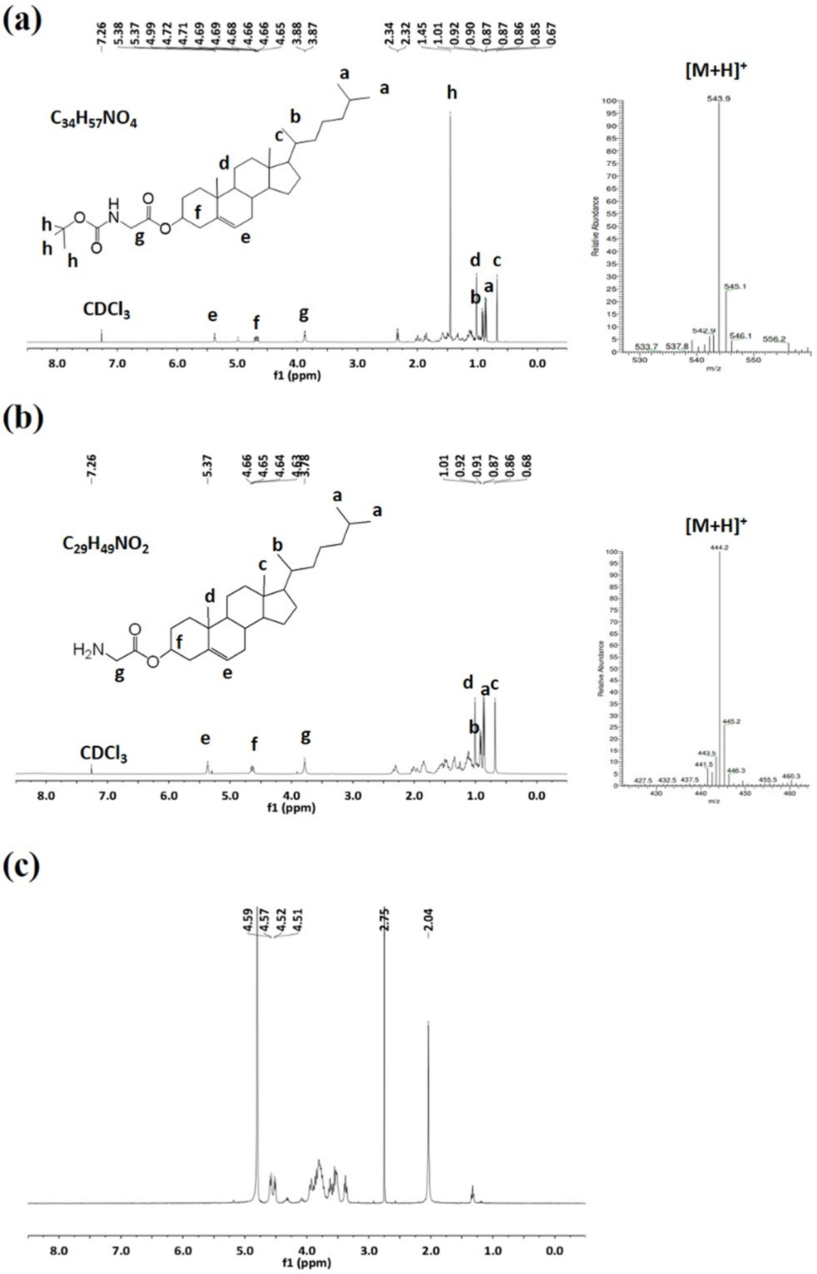

Figure S1. Structural identification: ^{1}H NMR ($CDCl_3$, 400 MHz) and mass spectrums (ESI-MS) of (a) Boc-Gly-cholesterol (b) NH_2-Gly-cholesterol; ^{1}H NMR spectrum (D_2O/d6-DMSO, 400 MHz) of (c) HACH20.

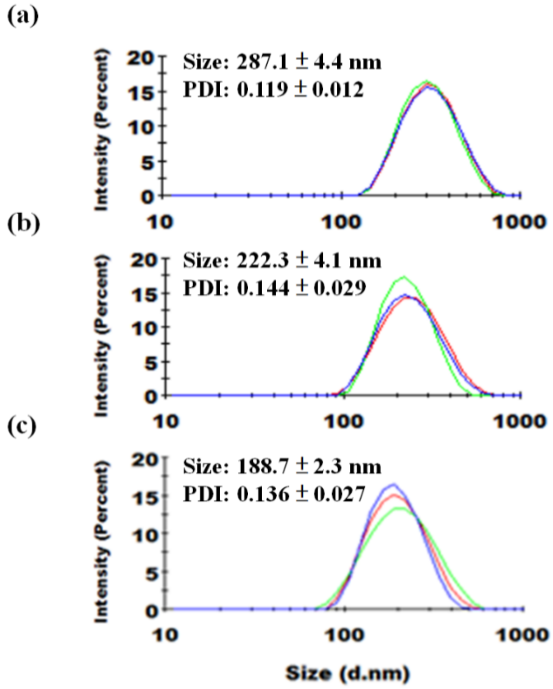

Figure S2. Size distribution of SQ@HACH with different passages: **(a)** 1 passage, **(b)** 2 passages, and **(c)** 3 passages through a microfluidizer. The size of SQ@HACH was measured under 100-fold dilution in water by DLS. (n = 3, mean ± SD).

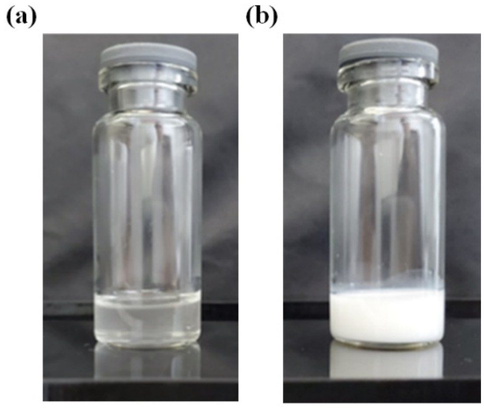

Figure S3. Appearance of **(a)** SQ@HA and **(b)** SQ@HACH. 5% squalene was added to the solution containing 1% HA and 1% HACH, homogenized and stood for 1 h.
